# Health insurance financing and patient retention in care at diabetics and hypertension clinics in Dar es Salaam and Pwani regions, Tanzania. A cohort study

**DOI:** 10.1371/journal.pgph.0002972

**Published:** 2024-03-07

**Authors:** Harrieth Manisha, Candida Moshiro, Ally Hussein, Fredrick Amani, Johnson Mshiu, Jaffar Shabbar, Sayoki Mfinanga

**Affiliations:** 1 National Institute for Medical Research, Dar es Salaam, Tanzania; 2 Tanzania Field Epidemiology and Laboratory Training Program, Dar es Salaam, Tanzania; 3 Department of Epidemiology and Biostatistics, School of Public Health, Muhimbili University of Health and Allied Sciences, Dar es Salaam, Tanzania; 4 UCL Institute for Global Health, London, United Kingdom; T D Medical College, INDIA

## Abstract

Hypertension and diabetes are chronic conditions that cause major morbidity and mortality worldwide. Whether health insurance financing is associated with improved retention in chronic care in Tanzania, is unknown. Our study establishes the effect of health insurance on retention and the determinants for retention in care among patients attending diabetes and hypertension clinics. We used a Cohort design to study participants enrolled in a cluster-randomized trial of integrated management of HIV, diabetes, and hypertension compared with standard vertical care in the INTE-AFRICA trial. Fifteen health facilities in Dar es Salam and Pwani regions were enrolled, with 1716 participants. Our sample size had 95% power to detect a 50% to 60% retention difference between the insured and uninsured groups (95% CI). We compared proportions using χ2 tests and obtained prevalence and rate ratios by Generalised Linear Models. We studied 1716 participants for 1612.3 Person-years (PY). At the study’s end, 1351 persons were alive and retained in care. Among the insured participants (26.0%), females accounted for 65.9%. Middle-aged adults contributed 58.8% of insured participants. We observed high retention rates (retention incidence rate IR: 83.80/100 PY; 95% CI (79.40–88.40)). There was no difference in retention among insured and uninsured patients (adjusted rate ratio aRR: 1.00; 95% CI, 0.94–1.06). Being middle-aged or senior-aged adults compared to young adults, having diabetes alone or hypertension alone compared to both conditions, having the comorbidity of diabetes or hypertension with HIV compared to a single condition, and attending health centres and hospitals compared to dispensaries were significantly associated with retention in care. This study showed no effect of health insurance on retention in diabetic and hypertension care clinics. However, age, medical diagnosis, morbidity, and type of health facility attended were associated with retention in care.

## Introduction

Non-communicable diseases (NCDs) account for more than 41 million deaths worldwide, with cardiovascular diseases (CVD) contributing about 18 million deaths annually. Hypertension is a major risk factor for CVD, affecting approximately 1 billion people globally. It is estimated that hypertension accounts for about 10 to 20 million cases among adults and up to 11% of adult deaths in Sub-Saharan Africa annually [1–3]. Diabetes is a risk factor for CVD, affecting approximately 537 million adults globally, and contributing to about 6.7 million deaths annually. Africa alone contributes about 24 million cases and about 416000 deaths annually [4]. In Tanzania, the prevalence of diabetes and hypertension among adult Tanzanians aged 24–64 years was reported to be about 9.1% and 25.9%, respectively [5].

Retention in care is essential for patients requiring regular lifelong care [6, 7]. The retention rates for hypertension and Diabetes patients in care are estimated to be only about 5 to 30% in sub-Saharan Africa [8]. There are many reasons for this, but lifelong care is unaffordable for many patients and may result in catastrophic health expenditure, which in turn may affect retention in care.

Health insurance aims to provide financial protection through the collective pooling of health risks among members, thereby increasing the use of healthcare resources [9]. The objectives of health insurance financing align with the Universal health coverage (UHC) movement. The UHC aims to attain financial protection and improve health system performance for everyone [10]. In Tanzania, close to 80% of the population is not covered by health insurance. Three types of health insurance schemes are common in Tanzania: The National Health Insurance Fund (NHIF), the Community Health Fund (CHF), and Private insurance financing. It is estimated that less than 10% of the total population in Tanzania is enrolled in the NHIF scheme, and about 7% is enrolled in the CHF scheme. Private health insurance schemes cover an additional 1% of the total population [11, 12].

We have a limited understanding of the role of health financing in lifelong care in Africa and Tanzania. Our study aims to understand the role of health financing in connection to lifelong care. To the best of the authors’ knowledge, this is the first study that assessed the effect of health insurance on retention and the determinants of retention among patients attending diabetes and hypertension clinics in the Dar es Salaam and Pwani regions.

## Material and methods

### Study design and population

We used the cohort design to study participants attending diabetes and hypertension clinics in the Dar es Salaam and Pwani regions of Tanzania. Our study utilized the framework of a large-scale implementation trial, the INTE-Africa trial, with one arm of integrated care for HIV, hypertension, and diabetes and the standard of care arm at the health facilities in Tanzania. INTE-Africa was assessing the effectiveness of integrating services for diabetes, hypertension, and HIV infection to reduce the burden of CVD. We studied 15 health facilities that were selected from the two regions and stratified by: District Hospitals, Health Centres, and Dispensaries, including Not-for-profit health facilities. Health facilities were obtained by generating a list of health facilities that fulfil the stratification criteria and purposely sampling out the 15 health facilities. Health facilities that did not have dedicated diabetes or hypertension care were excluded.

### Eligibility criteria

All adults (≥18 years) diagnosed with hypertension or diabetes enrolled in the INTE-Africa project in the Dar es Salaam and Pwani regions from July 08, 2020, to April 30, 2021.

### Power calculations

Our final sample size was 1716, with 26% of the participants owning health insurance. The sample size includes all participants from the INTE-Africa trial who met our eligibility criteria. Using the 2-sample, 2-sided approach, our final sample had the power of 95% to detect the difference of 50% to 60% retention between the two insured and uninsured groups considering the probability of type one error (α) at 5%. We used the Chow et al. 2018 formula and the online OpenEpi software [13].

### Operational definitions

Key terms used during the analysis are defined below:

1. *Single condition* was defined as participants with only diabetes or hypertension diagnosis at baseline.
2. *Comorbidity* was defined as Participants both with diabetes and hypertension diagnoses at baseline.
3. *Comorbidity with HIV* was defined as Participants with diabetes or hypertension with HIV diagnoses at baseline.
4. *Multimorbidity* was defined as Participants with diabetes, hypertension, and HIV diagnoses at baseline.
5. *Retention* was defined as whether a patient had two or more completed clinic visits separated by three or more months during a 12-month observation period [7]. This comprised persons who were not dead at the end of the follow-up period. We adopted the Human Resources and Services Administration HIV/AIDS Bureau (HRSA HAB) performance measure for retention in HIV care.
6. *Insured* was defined as clients that owned a health insurance scheme to finance health care at baseline.
7. *Uninsured*, was defined as clients that did not own a health insurance scheme at baseline.
8. *Loss to follow-up was* defined as participants who defaulted to care such that their status was unknown by their end-of-study visit assessment.

#### Variable measurement

The dependent variable was retention in care. The independent variables included participants’ gender, date of birth, height, weight, occupation, marital status, education status, health insurance financing, HIV status, complications, hypertension and/or diabetes diagnosis, the health facility they attend, and integration status.

### Data collection

An electronic abstraction was used to collect social demographic, clinical, and laboratory information from the INTE-Africa electronic database. Data was accessed from the INTE-Africa database from May 27–29, 2022. Data collection during the INTE Africa study was done using electronic case report forms (CRF) linked to a Python-based database with in-built automated validation checks digitally formatted for use on electronic tablets. Paper case report forms were also available as a backup if the electronic data capture failed on a particular day. Collated data were checked for consistency regularly, and errors were feedback to the field for learning purposes. Data collection was scheduled to be minimally disruptive to regular procedures at the clinic. The data forms were administered while participants waited to be serviced by their healthcare provider or were in the queue to collect their medicines. All research assistants involved in Data collection and management were trained on Good Clinical Practice (GCP) principles. Data collection was done at baseline, at every medical visit, and at the end of the study at 12 months.

### Statistical analysis

Data analysis was performed using Stata version 14 (Stata Corp LP, College Station, TX, USA). Before the analysis, data preparation was undertaken to check for any missing values or outliers, label variables, and generate new variables, and categories. Body Mass Index (BMI (weight (kg)/height (m^2^)) was generated and categorized into groups of Underweight (<18.5), normal weight (18.5–24.9), overweight (25–29.9), and obese (>30.0). Age categories were also generated as well as the morbidity status. Morbidity categories included: Single condition, comorbidity of diabetes and hypertension (labelled as comorbidity), comorbidity with HIV, and multimorbidity. Age groups used for comparisons were as follows: The youth (<25 years), young adults (25–44 years), middle-aged adults (45–64 years), and senior age adults (65+ years). The age category (20–44 years) was also used to represent young adults.

Proportions were compared using the chi-square test (χ2). Retention is reported as proportions and retention rates per person-years. Rate ratios for retention were obtained using Poisson regression analyses. The strength of association was presented using both crude and adjusted prevalence and rate ratios with corresponding 95% confidence intervals (CIs). Factors for multivariable models were selected based on prior knowledge of possible association with retention [6, 14, 15]. The multivariable model also included other variables with *p*-value < 0.2 in bivariable models. Differences were considered statistically significant when the 95% CI of the prevalence and rate ratios did not include one in the multivariable analysis.

### Ethical considerations

Authorization to use the project data was obtained from the INTE-AFRICA project leadership. The protocol was submitted for ethical clearance at the MUHAS ethical review board (Ref. No.DA.282/298/01.C/). All participants’ information was de-identified before the analysis to ensure anonymity.

## Results

**Fig 1:**
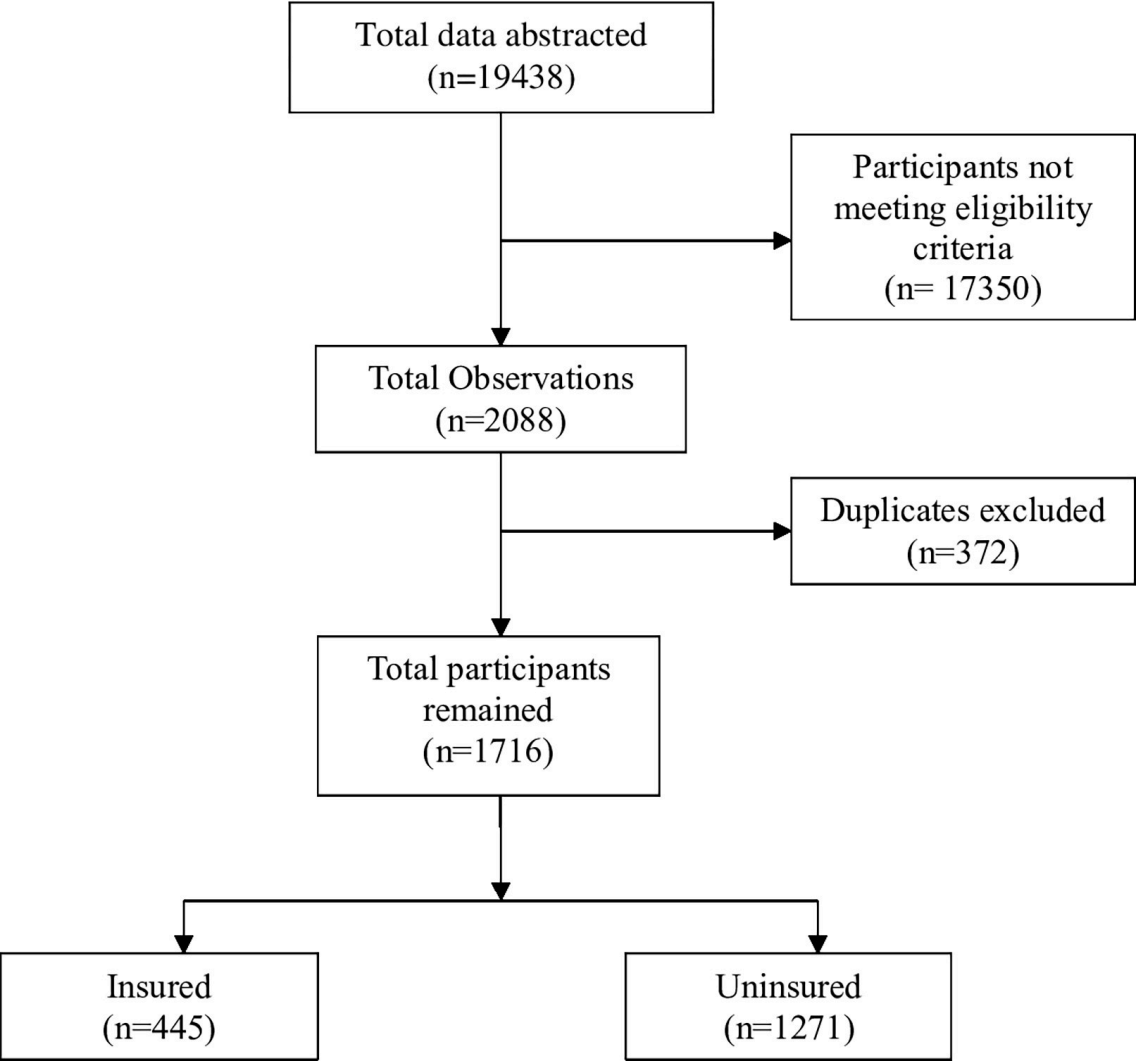
Flow chart of data abstracted from the INTE-Africa electronic database for patients enrolled from July 08, 2020, to April 30, 2021.

For the data flow chart, see Fig 1. ""Fig 1,"" shows data abstracted from the INTE-Africa electronic database for patients enrolled from July 08, 2020, to April 30, 2021.

### Baseline characteristics by insurance status

#### General characteristic

We included 1716 participants with diabetes and/or hypertension, of which 71.0% were female and 53.6% were aged 45–64. Married study participants were 57.7%. Participants with health insurance financing were 26.0%, among which insured females contributed 65.9% to total health insurance coverage. The age group 45–64 years had slightly above half (58.8%) of participants covered by health insurance, while those aged below 25 years were not covered at all. About two-thirds (63.4%) of Participants covered by the insurance were married (Table 1).

**Table 1:** General characteristics of patients attending diabetes and hypertension clinics in Dar es Salaam and Pwani regions from July 08, 2020, to April 30, 2021.

Variables	Total n (%)	Insured n (%)	Uninsured n (%)	*p-*value*
	(N = 1716)	N = 445	N = 1271	
**Gender**				
Male	498 (29.0)	151 (33.9)	347 (27.3)	0.008
Female	1218 (71.0)	294 (66.1)	924 (72.7)	
**Age groups years**				
<25	6 (0.3)	0 (0.0)	6 (0.5)	<0.001
25–44	220 (12.8)	26 (5.8)	194 (15.3)	
45–64	919 (53.6)	258 (58.0)	661 (52.0)	
65+	571 (33.3)	161 (36.2)	410 (32.3)	
**Body Mass Index**				
underweight	58 (3.4)	9 (2.0)	49 (3.9)	0.003
Normal weight	538 (31.4)	114 (25.7)	424 (33.4)	
Overweight	570 (33.2)	163 (36.7)	407 (32.0)	
Obese	549 (32.0)	158 (35.6)	391 (30.8)	
**Integration status**				
Standard	1006 (58.6)	265 (59.6)	741 (58.3)	0.645
Integrated	710 (41.4)	180 (40.4)	530 (41.7)	
**Type of Health Facility**				
Dispensary	185 (10.8)	20 (4.5)	165 (13.0)	<0.001
Health Centre	520 (30.3)	93 (20.9)	427 (33.6)	
Hospital	1011 (58.9)	332 (74.6)	679 (53.4)	
**Marital status**				
Single	58 (3.4)	8 (1.8)	50 (3.9)	0.003
Divorced	210 (12.2)	39 (8.8)	171 (13.5)	
Widowed	458 (26.7)	116 (26.1)	342 (26.9)	
Married	990 (57.7)	282 (63.4)	708 (55.7)	
**Education level**				
No formal education	369 (21.5)	63 (14.2)	306 (24.1)	<0.001
Primary	1069 (62.3)	252 (56.6)	817 (64.3)	
Secondary	221 (12.9)	91 (20.4)	130 (10.2)	
Tertiary	57 (3.3)	39 (8.8)	18 (1.4)	
**Employment status**				<0.001
Manual work	548 (31.9)	110 (24.7)	437 (34.5)	
Unemployed	195 (11.4)	44 (9.9)	151 (11.9)	
Housewife	366 (21.3)	63 (14.2)	303 (23.8)	
Other	66 (3.8)	14 (3.1)	52 (4.1)	
Retired	269 (15.7)	111 (24.9)	158 (12.4)	
Professional	272 (15.9)	103 (23.1)	169 (13.3)	

*The reported *p*-value is from the Chi-squared test.

#### Clinical characteristics

About two-thirds of participants (60.6%) had hypertension, while about one-fifth (17.8%) had diabetes alone. Participants with single morbidity had the highest proportion (59.8%), and those with multimorbidity had the lowest proportion (2.9%). Few participants had no complications (38.1%). Out of all participants, 55.3% (445) of those insured had hypertension, and 54.6% (445) of those insured were diagnosed with a single condition (either hypertension or diabetes) (Table 2).

**Table 2:** Clinical characteristics of patients attending diabetes and hypertension clinics in Dar es Salaam and Pwani regions from July 08, 2020, to April 30, 2021.

Variables	Total n (%)	Insured n (%)	Uninsured n (%)	*p*-value *
	(N = 1716)	N = 445	N = 1271	
**Medical Condition**				
Diabetes alone	305 (17.8)	60 (13.5)	245 (19.3)	<0.001
Hypertension and Diabetes	371 (21.6)	139 (31.2)	232 (18.3)	
Hypertension alone	1040 (60.6)	246 (55.3)	794 (62.5)	
**Morbidity**				
Single Condition	1027 (59.8)	243 (54.6)	784 (61.7)	<0.001
Comorbidity with HIV	319 (18.6)	66 (14.8)	253 (19.9)	
Comorbid with DM** or HTN***	321 (18.7)	118 (26.5)	203 (16.0)	
Multimorbidity	49 (2.9)	18 (4.0)	31 (2.4)	
**Complications**				
No	654 (38.1)	161 (36.1)	493 (38.8)	0.006
Stroke	22 (1.3)	7 (1.6)	15 (1.2)	
Heart Attack	23 (1.3)	8 (1.8)	15 (1.2)	
Renal disease	4 (0.2)	4 (0.9)	0 (0.0)	
Vision	251 (14.6)	70 (15.7)	181 (14.2)	
Numbness	286 (16.7)	60 (13.5)	226 (17.8)	
foot ulcers	11 (0.6)	2 (0.4)	9 (0.7)	
Duo Complication	381 (22.2)	104 (23.4)	277 (21.8)	
Multi-complication	84 (4.9)	29 (6.5)	55 (4.3)	

*The reported *p*-value is from the Chi-squared test

**DM- Diabetes

***HTN- Hypertension.

### Patient follow-up outcomes by insurance status

#### Follow-up description by insurance status

Each participant was followed up for at least 12 months, and the last follow-up was on April 29, 2022. The median follow-up time in days was 370, with an IQR of 42. A total of 445 (26.0%) participants were insured. During follow-up, only 0.9% (15) of the participants were withdrawn, 0.06% (1) did not meet the study enrolment criteria, 3.2% (55) were lost to follow-up, and 3.5% (60) died during the study (Table 3).

**Table 3:** Follow-up outcomes for patients attending diabetes and hypertension clinics in Dar es Salaam and Pwani regions from July 08, 2020 to April 30, 2022.

Variables	Total	Insured	Uninsured
Number enrolled	1716	445	1271
Number withdrawn, n (%)	15 (0.9)	5 (1.1)	10 (0.8)
Late Exclusion due to ineligibility n (%)	1 (0.1)	1 (0.2)	0 (0.0)
Number died, n (%)	60 (3.5)	19 (4.3)	41 (3.3)
Number Transferred out, n (%)	122 (7.1)	44 (10.0)	78 (6.2)
Number LTFU*, n (%)	55 (3.2)	8 (1.8)	47 (3.7)
Median follow-up time Days (IQR**)	370 (42)	366 (39)	371 (44)

*LTFU–Loss to follow-Up

**IQR–Interquartile Range.

#### Proportions of loss to follow-up and retention at 6 and 12 months

The proportions of retention at six months were (p: 0.81; 95% CI, 0.73–0.90) among the insured and (p: 0.82; 95% CI, 0.77–0.87) among the uninsured. The cumulative incidence for retention at twelve months was (p: 0.80; 95% CI, 0.71–0.88) among the insured and (p: 0.79; 95% CI, 0.75–0.85) among the uninsured (Table 4).

**Table 4:** Proportion of loss to follow-up and retention in care at 6 and 12 months among patients attending diabetes and hypertension clinics in Dar es Salaam and Pwani regions from July 08, 2020, to April 30, 2022 (N = 1716).

Variables	Total (N = 1716)	Insured (N = 445)		Uninsured (N = 1271)	
		Number	Proportion, (95% CI*)	Number	Proportion, (95% CI*)
LTFU** at 6 months	39	4	0.01 (0.00–0.02)	35	0.03 (0.02–0.03)
LTFU** at 12 months	55	8	0.02 (0.01–0.04)	47	0.04 (0.03–0.05)
Retention at 6 months	1386	357	0.80 (0.72–0.89)	1029	0.81 (0.76–0.86)
Retention at 12 months	1351	350	0.79 (0.71–0.87)	1001	(0.74–0.84)

*CI–Confidence Interval

**LTFU–Loss to Follow-Up

### Retention rates among insured and uninsured patients at 12 months

Among 1716 participants followed up for 1612.3 Person Years (P-Y), 1351 were alive and retained, making an overall retention incidence rate (IR) = 83.80/100 P-Y; 95% CI (79.40–88.40). Among the insured group, the retention rate was IR = 84.40/100P-Y; 95% CI (78.56–88.93); in uninsured patients, the retention rate was IR = 83.58/100P-Y; 95% CI (76.01–93.73). There was no significant difference in the retention rates of insured patients and uninsured patients (adjusted Rate Ratio (aRR: 1.00; 95% CI, 0.94–1.06). See Table 5.

**Table 5:** Bivariable and multivariable analysis of determinants for retention among patients attending diabetes and hypertension clinics in Dar es Salaam and Pwani regions from July 08, 2020, to April 30, 2022.

Variables	Number retained / person-year	Retention rate/ 100 person-year (95% CI*)	Unadjusted RR** (95% CI*)	Adjusted RR** (95% CI)
**Insurance status**				
Uninsured	1001/1197.6	83.58 (78.56–88.93)	REF	REF
Insured	350/414.7	84.40 (76.01–93.73)	1.01 (0.89–1.14)	1.00 (0.94–1.06)
**Gender**				
Male	371/459.7	80.71 (72.90–89.35)	REF	REF
Female	980/1152.6	85.03 (89.87–90.53)	1.08 (0.96–1.22)	1.05 (0.98–1.13)
**Age groups years**				
20–44	162/212.5	76.24 (65.36–88.93)	REF	REF
45–64	735/873.7	84.13 (78.26–90.44)	1.1 (0.93–1.31)	1.10 (1.01–1.20)
65+	454/526.1	86.29 (78.71–94.61)	1.1 (0.92–1.32)	1.16 (1.05–1.28)
**Body Mass Index**				
Normal weight	415/497.9	83.35 (75.70–91.76)	REF	REF
underweight	39/51.3	76.05 (55.56–104.09)	0.87 (0.63–1.21)	0.87 (0.73–1.03)
Overweight	454/540.9	83.92 (76.55–92.01)	1.03 (0.90–1.18)	1.02 (0.96–1.09)
Obese	443/521.0	85.02 (77.46–93.32)	1.05 (0.92–1.20)	1.02 (0.96–1.09)
**Medical Conditions**				
Hypertension and Diabetes	289/347.9	83.07 (74.02–93.22)	REF	REF
Diabetes alone	225/286.4	78.56 (68.94–89.53)	0.95 (0.80–1.13)	1.19 (1.07–1.32)
Hypertension alone	837/977.9	85.59 (79.98–91.59)	1.03 (0.90–1.18)	1.24 (1.14–1.34)
**Morbidity**				
Single condition	774/954.5	81.09 (75.57–87.00)	REF	REF
Comorbidity HIV	288/309.0	93.21 (83.04–104.62)	1.20 (1.05–1.37)	1.20 (1.13–1.26)
Comorbidity DM & HTN	245/302	81.00 (71.47–91.81)	1.01 (0.88–1.17)	1.13 (0.46–2.79)
Multimorbidity	44/46.3	95.73 (70.73–127.72)	1.19 (0.88–1.61)	1.37 (0.56–3.4)
**Integration status**				
Standard of Care	822/963.0	85.36 (79.72–91.40)	REF	REF
Integrated	529/649.3	81.47 (74.82–88.72)	0.91 (0.82–1.02)	0.90 (0.85–0.95)
**Type of Health Facility**				
Dispensary	129/170.4	75.72 (63.72–89.98)	REF	REF
Health Centre	426/489.4	87.05 (79.16–95.72)	1.17 (0.96–1.43)	1.13 (1.02–1.26)
Hospital	796/952.5	83.57 (77.96–89.58)	1.13 (0.94–1.36)	1.12 (1.01–1.24)
**Marital status**				
Single	52/55.1	94.33 (71.88–123.79)	REF	REF
Divorced	175/199.7	87.63 (75.56–101.62)	0.93 (0.68–1.27)	0.86 (0.77–0.96)
Widowed	372/429.5	86.61 (78.24–95.87)	0.91 (0.68–1.21)	0.85 (0.77–0.95)
Married	752/927.9	81.04 (75.45–87.05)	0.85 (0.64–1.12)	0.85 (0.77–0.93)
**Education level**				
No formal education	292/338.0	86.38 (77.02–96.88)	REF	REF
Primary	852/1013.9	84.03 (78.57–89.87)	1.01 (0.88–1.15)	1.00 (0.93–1.06)
Secondary	163/208.8	78.06 (69.95–91.01)	0.93 (0.77–1.13)	0.93 (0.84–1.03)
Tertiary	44/51.5	85.41 (63.56–114.77)	0.98 (0.71–1.34)	0.96 (0.83–1.12)
**Employment status**				
Other	52/62.9	82.66 (62.98–108.47)	REF	REF
Unemployed	156/178.5	87.37 (74.68–102.22)	1.02 (0.74–1.39)	0.99 (0.86–1.16)
Retired	207/247.1	83.76 (73.09–95.98)	0.98 (0.72–1.32)	1.03 (0.89–1.20)
Professional	217/261.7	82.91 (72.58–94.71)	1.01 (0.75–1.37)	1.02 (0.88–1.17)
Housewife	292/348.5	83.79 (74.71–93.97)	1.01 (0.75–1.36)	1.01 (0.87–1.16)
Manual work	427/313.4	83.16 (75.64–91.44)	0.99 (0.74–1.32)	1.01 (0.88–1.16)

*CI–Confidence Intervals

**RR–Rate Ratio.

### Determinants for retention at 12 months follow-up

In the multivariable analysis, the retention rates were associated with age, medical condition, morbidity status, health facility integration status, type of health facility, and marital status as follows: Middle-aged adults and senior-aged adults 1.10 (95% CI, 1.01–1.20) and 1.16 (95% CI, 1.05–1.28) times more likely to be alive and retained in care compared to young adults. Participants with diabetes alone or hypertension alone were 1.19 (95%, 1.07–1.32) and 1.24 (95% CI, (1.14–1.34) times more likely to be alive and retained in care when compared to those with both diabetes and hypertension conditions, respectively. Patients with comorbidity of diabetes or hypertension with HIV were 1.20 (95% CI, 1.13–1.26) and 1.46 (95% CI, 1.28–1.67) times more likely to be alive and retained in care compared to those with single conditions, respectively. Participants attending clinics at health centres and hospitals were 1.13 (95% CI, 1.02–1.25) and 1.12 (95% CI, 1.01–1.24) times more likely to be alive and retained in care than those attending dispensaries, respectively. Divorced, widowed, and married participants were participants 0.86 (95% CI, 0.77–0.96), 0.85 (95% CI, 0.77–0.95), and 0.84 (95% CI, 0.77–0.93) times less likely to be alive and retained care compared to single respectively (Table 5).

## Discussion

### Retention for patients attending diabetes and hypertension clinics

Our study observed high retention rates (83.80/100 Person Years) for patients attending diabetes and hypertension clinics. This differed from other studies reporting low retention rates for patients with diabetes and /or hypertension in Africa [16]. The observed high retention rates may have been an influence of the COVID pandemic, which has encouraged care-seeking behaviour, especially among people with chronic conditions, and thus affected our findings. Furthermore, the study participants were aware of being followed up, potentially influencing the observed findings. Also, training was provided to care providers in all facilities enrolled in the trial. However, we must highlight that the trial exclusively introduced the integration model, keeping all other interventions, procedures, and financing unchanged. All costs related to medical visits including consultation fees, laboratory tests, and drugs were required to be covered by the patient through direct out-of-pocket, health insurance, or exemption through government aid programs [17].

On the other hand, the trial allowed consistent data collection and storage which facilitated easier access to follow-up data, suggesting that retention rates may have consistently been high. Additionally, the high retention rates observed in our study settings emphasize the need for further investigations to determine if this pattern persists. The high rates for retention were similar to another study in Africa with an integrated model approach [18].

Our study revealed that only around 26.0% of insured adult patients attended diabetes and hypertension clinics. While this was slightly higher than the reported health insurance rate in the general population (estimated to be less than 20%), it falls short of aligning with the Universal Health Coverage (UHC) emphasis on equitable benefits [11, 19].

Contrary to our hypothesis, our study revealed no significant effect of health insurance on retention in care. We anticipated that patients utilizing health insurance financing would exhibit better retention compared to uninsured patients. While we couldn’t find relevant studies on individuals receiving diabetes and/or hypertension care, we did compare our findings with a study on women living with HIV in the USA. Interestingly, unlike our study, this comparison showed that health insurance financing was associated with better retention in care [20].

### Determinants for retention in care among patients attending diabetes and hypertension clinics

Our study findings have shown that; age, medical diagnosis, morbidity status, type of health facility, and marital status were associated with retention in care among our study participants. On the other hand, sex employment and BMI were not associated with retention in care.

Middle-aged and senior adults are known to assume more risk for their medical conditions, are probably less mobile, and able to afford the costs associated with retention to care and there hence, our observed results [21–23].

Participants with the comorbidity of diabetes or hypertension with HIV were more likely to be alive and retained to care compared to those with single conditions of diabetes or hypertension due to possible influences of HIV and the HIV program. Participants with HIV are known to assume more risk to their conditions, and the HIV program tends to impact care-seeking habits among these patients [24].

Attending clinics at health centres and hospitals was observed to be associated with retention in care. Health centres and hospitals may offer more service options, including specialists and even a more comprehensive range of drugs and treatments.

Our study has shown that being divorced, widowed, and married decreased the likelihood of retention in care when compared to being single. This is contrary to what is expected, as single people tend to be more mobile and less economically stable [21, 22].

The findings from our study have shown that sex employment and BMI were not associated with retention in care, and these findings were similar to the findings from a study done in Malawi on accessing clinical services following screening for hypertension [8].

### Study strengths and limitations

To our knowledge, this is one of the first studies investigating the retention in care and the effect of health insurance financing on retention for patients attending diabetes and hypertension clinics in Tanzania. The INTE-Africa trial provided us with a large sample size and good-quality data. Our findings should be interpreted while considering the following limitations: The study used health facilities selected from only two regions: Dar es Salaam and Pwani, which are all located on the coast; hence the data may not be used to generalize the whole country. However, the selected facilities covered all types of health facilities in Tanzania, including District hospitals, Health Centres, Dispensaries, and Not-for-profit health facilities. Furthermore, the data utilized in this study was collected to assess the effectiveness of integrating services for diabetes, hypertension, and HIV/AIDS in reducing the burden of cardiovascular disease within the INTE-Africa trial. It was not explicitly aimed at addressing the objectives of our study.

## Conclusion

Retention in care was not associated with health insurance financing; however, medical diagnosis, comorbidity with HIV, health facility integration, the type of health facility, and marital status were significantly associated with retention in care. However, with the low health insurance financing observed in our study, we call for the government of Tanzania and other stakeholders to join forces in making health insurance available and accessible for all while moving towards UHC. Further, we recommend more research in the area of health financing and lifelong care.

## Supporting information

S1 Data Patients enrolled in the INTE-Africa electronic database from July 08, 2020, to April 30, 2021. (XLSX)
